# Multiple signaling pathways regulate contractile activity‐mediated PGC‐1*α* gene expression and activity in skeletal muscle cells

**DOI:** 10.14814/phy2.12008

**Published:** 2014-05-20

**Authors:** Yuan Zhang, Giulia Uguccioni, Vladimir Ljubicic, Isabella Irrcher, Sobia Iqbal, Kaustabh Singh, Shuzhe Ding, David A. Hood

**Affiliations:** 1 School of Kinesiology and Health Science, and Muscle Health Research Centre, York University, Toronto, M3J 1P3, Ontario, Canada; 2 Department of Sport and Health Science, Nanjing Sport Institute, Nanjing, China; 3 Key Laboratory of Adolescent Health Assessment and Exercise Intervention, Ministry of Education of China, East China Normal University, Shanghai, China

**Keywords:** AMP kinase, calcium signaling, exercise, mitochondrial biogenesis, p38 MAP kinase, reactive oxygen species, skeletal muscle

## Abstract

PGC‐1*α* is an important transcriptional coactivator that plays a key role in mediating mitochondrial biogenesis. Within seconds of the onset of contractile activity, a number of rapid cellular events occur that form part of the initial signaling processes involved in PGC‐1*α* gene regulation, such as elevations in cytoplasmic calcium, AMPK and p38 activation, and elevated ROS production. We observed that basal levels of PGC‐1*α* promoter activity were more sensitive to resting Ca^2+^ levels, compared to ROS, p38 or, AMPK signaling. Moreover, enhanced PGC‐1*α* transcription and post‐translational activity on DNA were a result of the activation of multiple signal transduction pathways during contractile activity of myotubes. AMPK, ROS, and Ca^2+^ appear to be necessary for the regulation of contractile activity‐induced PGC‐1*α* gene expression, governed partly through p38 MAPK and CaMKII activity. Whether these signaling pathways are arranged as a linear sequence of events, or as largely independent pathways during contractile activity, remains to be determined.

## Abstract (precis)

When we exercise regularly, one of the most pronounced adaptations is the increase in mitochondrial content in skeletal muscle. This adaptation allows us to exercise longer with less fatigue, and it stimulates to breakdown of fat as an energy source. In this study, we sought to investigate some of the molecular signals which underlie this adaptation. Using a cell culture model of muscle contractile activity, we investigated the expression and regulation of one of the most important proteins that controls mitochondria: PGC‐1alpha. This protein is a transcription factor that regulates the expression of numerous genes which mediate the synthesis of mitochondria. Our results indicate that there are several redundant signaling pathways, activated by exercise, that control the transcription and activity of PGC‐1alpha. This is likely beneficial to insure that exercise adaptations in muscle can take place, even if one pathway is deficient.

## Introduction

The peroxisome proliferator‐activated receptor‐*γ* (PPAR*γ*) coactivator 1*α* (PGC‐1*α*) is currently considered to be a dominant regulator of mitochondrial function, respiration, and biogenesis in skeletal muscle (Wu et al. 1999; Vega et al. 2000; Kelly and Scarpulla 2004). It was first discovered as an interacting partner with PPAR*γ*, and its expression was found to be greatly up‐regulated in brown fat in response to cold exposure (Puigserver et al. 1998). PGC‐1*α* is able to regulate mitochondrial content largely due to its capacity to coactivate multiple transcription factors, more specifically genes involved in the regulation of nuclear genes encoding mitochondrial proteins (NUGEMPs). PGC‐1*α* is also important for mediating contractile activity‐induced mitochondrial adaptations (Geng et al. 2010; Uguccioni and Hood 2011), particularly during the aging process (Leick et al. 2010). Skeletal muscle has an exceptional capability to adapt to exercise, and the molecular basis for these adaptive responses is currently under intensive investigation (Egan and Zierath 2013). An important part of understanding these molecular adaptations is a comprehension of the signaling cascades which converge to control PGC‐1*α* mRNA and protein content (Irrcher et al. 2003a; Pilegaard et al. 2003; Jager et al. 2007). Indeed, multiple signaling pathways become activated in response to muscular contractions, helping to regulate PGC‐1*α* levels. The two primary protein kinases involved in the regulation of PGC‐1*α* within skeletal muscle are AMPK (Jager et al. 2007) and p38*γ* MAPK (Aronson et al. 1997; Puigserver et al. 2001; Akimoto et al. 2005; Pogozelski et al. 2009). Previous work has demonstrated that the activation of AMPK (Irrcher et al. 2008) and p38 induces PGC‐1*α* mRNA expression and transcriptionally activate its promoter. In addition, this transcription is influenced by cross‐talk with other pathways. For example, the increase in PGC‐1*α* induced by raising cytosolic Ca^2+^ is mediated by CAMKII, the main CAMK isoform present in skeletal muscle. It is speculated that CAMKII functions as an upstream kinase during exercise to alter PGC‐1*α* expression through the regulation of p38 (Rose et al. 2006; Wright 2007; Wright et al. 2007) and AMPK activation (Woods et al. 2005; Jense et al. 2007). Alternatively, reactive oxygen species (ROS) are also involved in p38 MAPK and AMPK activation, and the subsequent regulation of PGC‐1*α* transcriptional activity and expression (Irrcher et al. 2009; Kang et al. 2009). Ca^2+^ and ROS can also participate in the activation of protein kinases, which in turn phosphorylate downstream targets, such as transcription factors.

The purpose of this study was to use a muscle cell culture system to investigate the role of divergent contractile activity‐induced intracellular signaling pathways on the transcriptional activation of the mouse PGC‐1*α* promoter. In particular, we were interested in uncovering how exercise‐induced PGC‐1*α* expression is regulated in response to upstream signals, such as elevations in cytoplasmic (Ca^2+^), cross‐bridge cycling, ATP turnover, and ROS production.

## Materials and Methods

### Cell culture and treatment

C2C12 murine skeletal muscle cells (ATCC, Manassas, VA) were cultured as previously described (Connor et al. 2001; Uguccioni and Hood 2011). Briefly, cells were proliferated on six‐well culture dishes (Sarstedt, Montreal, QC) coated with 0.1% gelatin (Sigma, St. Louis, MO) in Dulbecco's modified Eagle's medium (DMEM) (Sigma) supplemented with 10% fetal bovine serum (FBS; Fisher Scientific, Ottawa, ON) and 1% penicillin‐streptomycin (P/S) (Invitrogen, Carlsbad, CA). At 80–90% confluence, differentiation into myotubes was induced by switching the medium to DMEM supplemented with 5% heat‐inactivated horse serum (HS) (Invitrogen) and 1% P/S. On days 5–6 of differentiation, cells were treated with either vehicle (H_2_O or DMSO) or 20 mmol/L N‐acetylcysteine (NAC), 40 *μ*mol/L Compound C (CC), 100 *μ*mol/L BAPTA‐AM, 150 *μ*mol/L N‐benzyl‐p‐toluene sulfonamide (BTS), or 10 *μ*mol/L BIRB796 (BIRB) for 30 or 60 min (NAC). Drug treatments were followed by acute electrical stimulation (2 h; 5 Hz; 10V) to induce contractile activity (CA) of the myotubes in vitro, as previously described in detail (Connor et al. 2001; Uguccioni and Hood 2011) using a custom‐made stimulator and six‐well plate lids outfitted with two platinum wires per well which were submerged into the media of plates containing fully differentiated myotubes. Cells were scraped immediately after the CA period, or were allowed to recover for either 2 or 24 h, as specified in the Results.

### Transfections and dual‐luciferase assay

(1) PGC‐1*α* promoter activity: C2C12 myoblasts were transiently transfected with 2 µg/well of the 2 kb mouse PGC‐1*α* promoter plasmid (Addgene) linked to the luciferase reporter gene pGL3. Transfection efficiency was normalized to *Renilla* luciferase activity (pRL‐CMV; 5 ng/plate); (2) PGC‐1*α* activity: C2C12 myoblasts were cotransfected with a chimeric gene encoding full‐length PGC‐1*α* fused to the DNA‐binding domain (DBD) of GAL4 of pBind vector (0.05 µg/well) as previous described (Puigserver et al. 1998, 1999), and a luciferase construct driven by five GAL4 DBD consensus sequences, referred to as pG5‐Luciferase vector (3 µg/well). Myoblasts were also cotransfected with a control pBind vector solely expressing the DBD of GAL4. On the same day (6 h post transfection), differentiation was initiated by switching the cells to differentiation medium. Following 5–6 days of differentiation, C2C12 myotubes were subjected to 2 h of electrical stimulation‐evoked CA, and enzyme extracts were made immediately, or following the recovery period. Luciferase activities were measured using a Lumat LB9507 luminometer (EG&G Berthold) according to the manufacturer's instructions.

### RNA isolation and RT‐PCR analyses

RNA was isolated from C2C12 muscle cells as described previously (Connor et al. 2001) with TRIzol reagent and resuspended in 25 *μ*L of sterile water. Total RNA concentration and purity were assessed using absorbance readings at 260 and 280 nm. Reverse transcription was performed using Superscript II Reverse Transcriptase (Invitrogen) in accordance with the instructions of the manufacturer, and PCR experiments were conducted using the following primers [PGC‐1*α*: 5′‐GAC CAC AAA CGA TGA CCC TCC‐3′(F) and 5′‐GCC TCC AAA TCT CTC AGG‐3′(R); *β*‐actin: 5′‐ACT GAC TAC CTC ATG AAG AT‐3′(F) and 5′‐CGT CAT ACT CCT GCT TGC TGA T‐3′(R)]. PCR products were analyzed on ethidium bromide‐stained agarose gels, and the expected size products were quantified using Sigma Scan Pro (ver. 5; Jandel Scientific, San Rafael, CA) software. The quantification of all target genes was corrected using the internal control *β*‐actin.

### Cellular Fractionation

Myotubes were harvested in ice‐cold PBS (Sigma) and subjected to cellular fractionation using the NE‐PER Nuclear and Cytoplasmic Extraction Kit as described by the manufacturer (Thermo Fisher Scientific, Rockford, IL)

### Immunoblotting

Total protein was isolated from C2C12 myotubes as done previously (Uguccioni and Hood 2011). Briefly, equal amounts of protein extracts (30–50 *µ*g) were separated on 8–12% SDS‐polyacrylamide gels and transferred to nitrocellulose membranes (GE Healthcare, Waukesha, WT). Membranes were blocked for 1 h with 5% skim milk or BSA in 1 × TBST solution (Tris‐buffered Saline/Tween‐20; 25 mmol/L Tris‐HCl, pH 7.5, 1 mmol/L NaCl and 0.01% Tween‐20), and subsequently probed overnight at 4°C with primary antibodies directed toward PGC‐1*α* (1:500; Millipore, Billerica, MA), phospho‐AMP‐activated protein kinase (AMPK; 1:200; Thr^172^; Cell Signaling, Danvers, MA), total AMPK*α* (1:1000; Cell Signaling), phospho‐acetyl‐CoA carboxylase (ACC; 1:500; Ser79; Cell Signaling), phospho‐p38 (1:200; Thr180/Thr182; Cell Signaling), total p38 (1:1000; Cell Signaling), Phospho‐calcium/calmodulin‐dependent protein kinase II (1:1000; Thr286; Cell Signaling), Histone H2B (1:1000; Cell Signaling), and *α*‐Tubulin (1:8000; Millipore). Subsequently, membranes were washed (3 × 5 min) using 1 × TBST and incubated with horseradish peroxidase‐conjugated secondary antibodies for 1 h at room temperature. Membranes were developed with Western Blot Luminol Reagent (Santa Cruz Biotechnology, Santa Cruz, CA). Films were scanned and analyzed using Sigma Scan Pro software. The quantification of all blots was corrected for loading using *α*‐tubulin or aciculin.

### Immunofluorescence

C2C12 myotubes were grown on custom‐made glass‐bottom six‐well dishes. Myotubes were washed with PBS, fixed in 3% buffered paraformaldehyde for 7 min at room temperature, followed by 5 mins of incubation with cold permeabilizing solution (0.1% Triton X‐100, 200 mmol/L Sucrose, 50 mmol/L Tris‐HCl (pH7.4), 5 mmol/L MgCl_2_). Subsequently, myotubes were placed in blocking solution (PBS containing 10% goat serum and 2% BSA), and immunostained (overnight at 4°C) with primary antibodies: PGC‐1 (H‐300; 1:200, Santa Cruz). After washing with PBS, cells were further incubated (1 h at room temperature) with Alexa 488 (1:1000, Invitrogen), rinsed, and mounted with anti‐bleaching/mounting reagent (2.5% DABCO, 10% Polyvinyl alcohol, 5% Glycerol, 25 mmol/L Tris, pH 9.0). Counterstaining was performed with DAPI (1:1000) to identify nuclei. Cells were then examined using Nikon Eclipse TE2000‐U microscope, and all images were taken at 60× magnification. The images presented are representative of approximately 35 images for each condition.

### Statistical analyses

All data are expressed as means ± SE. Where indicated, Student's unpaired *t*‐test or two‐way ANOVA were used to determine individual difference between conditions. Results were considered to be statistically significant if *P *<**0.05 was achieved.

## Results

### Contractile activity evokes PGC‐1α gene expression, transcriptional activity, and subcellular redistribution

**Figure 1.**
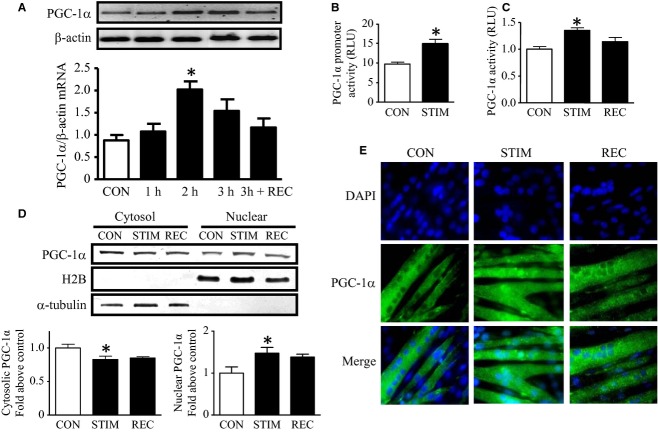
Induction of peroxisome proliferator‐activated receptor‐*γ* coactivator‐1 protein‐*α* (PGC‐1*α*) gene expression and translocation following acute contractile activity. (A) *Top*, total RNA extracts were prepared and PCR analyses were performed. Ethidium bromide (EtBr)‐stained DNA gel of PGC‐1*α* was amplified by PCR from control, stimulated (1 h, 2 h, and 3 h) and recovery (REC, 24 h post stimulation) cells. *β*‐actin was also amplified by PCR and used to verify equal loading. *Bottom*, graphical summary of repeated experiments of the effects of contractile activity on PGC‐1*α *mRNA expression (*n* = 6). (B) Acute contractile activity‐induced transcriptional regulation of the PGC‐1*α* promoter. Cells were transfected with the PGC‐1*α* luciferase constructs and harvested for the measurement of luciferase activities. PGC‐1*α* promoter activity in control and stimulated cells is shown (*n* = 6–8). RLU, relative light units. (C) Coactivation activity of PGC‐1*α* was measured using a chimeric gene encoding full‐length PGC‐1*α* fused to the DNA‐binding domain (DBD) of GAL4. Assessment of luciferase activity driven by GAL4 DBD consensus sequences (*n* = 6). REC, 2 h postcontractile activity. RLU, relative light units. (D) Representative Western blots are shown for each indicated protein and H2B or *α*‐tubulin (*top)*, with graphic quantification (*below*) expressed as fold change of PGC‐1*α* content in cytosol (*left*) and nuclear (*right*) fractions in response to different conditions (*n* = 4). A.U., arbitrary units. (E) Images of nuclear area (*blue*) using 10 *μ*mol/L of DAPI and PGC‐1*α* (*green*) used immunofluorescence with the antibody proven to be specific for PGC‐1*α* in control, stimulation, and recovery myotubes. All images were taken at 60× magnification. The images presented are representative of approximately 35 images for each condition. (*P < 0.05 vs. control cells). CON, control; STIM, stimulation; REC, recovery.

To assess alterations in PGC‐1*α* gene expression in response to acute contractile activity, PGC‐1*α* mRNA was examined at various durations of stimulation (STIM). As shown in Figure 1A, PGC‐1*α* mRNA was significantly enhanced in response to contractile activity and peaked at 2 h, as evident by a 2.5‐fold increase in mRNA expression. PGC‐1*α* mRNA returned to basal levels following 24 h of recovery. The changes in mRNA were mediated, at least in part, by transcriptional mechanisms, as PGC‐1*α* promoter activity was increased by 55% after 2 h of contractile activity. Acute contractile activity also augmented PGC‐1*α* protein activity, as documented by a 35% increase in PGC‐1*α*‐driven transcription of a luciferase reporter (Fig. 1B). These changes were reversed during the 2‐h recovery period (Fig. 1C). These rapid alterations in the regulation of PGC‐1*α* activity were matched by concomitant increases in PGC‐1*α* translocation to the nucleus as demonstrated by the expression profile in cytosolic and nuclear fractions, and by immunofluorescence staining (Fig. 1D,E).

### Multiple signaling kinases are activated with acute contractile activity

**Figure 2.**
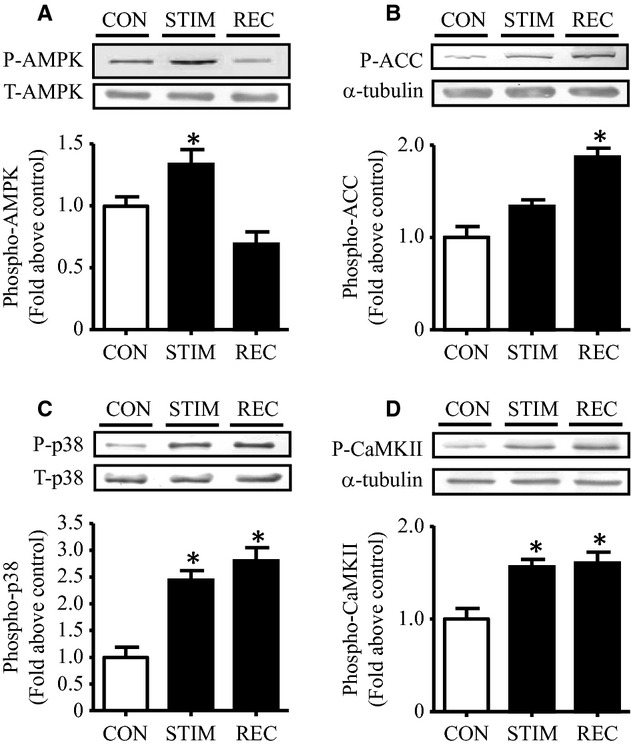
Phosphorylation of signaling kinases in response to acute contractile activity. *A‐D:* Phosphorylation of AMPK (A), ACC (B), p38 (C), and CaMKII (D) in C2C12 cells under control, stimulation, and recovery conditions. Representative Western blots were shown for each phosphorylated protein and its respective loading control (A–D, *top*), with graphical quantification (A–D, *bottom*) expressed as the fold change to control group (*n* = 6). A.U., arbitrary units. (*P < 0.05 vs. control cells). P, phosphorylated; T, total; CON, control; STIM, stimulation; REC, recovery.

We next sought to elucidate the signaling mechanisms involved in mediating the effect of contractile activity on PGC‐1*α* transcription and activity. AMPK and p38 mitogen‐activated protein kinase (MAPK) are two well‐recognized signaling kinases that influence PGC‐1*α* expression and mitochondrial content (Puigserver et al. 2001; Jager et al. 2007). As expected, contractile activity resulted in a significant increase in the phosphorylation of AMPK (Fig. 2A), and this activity was reflected in the phosphorylation of its downstream target ACC, evident during the 2‐h recovery period (Fig. 2B). In addition, contractile activity significantly augmented the phosphorylation of p38 (Fig. 2C) and p‐CaMKII (Fig. 2D), and their activation remained elevated during the recovery period. Thus, the timing of these events suggests that the phosphorylation and activation all three of these kinases (AMPK, p38, and CaMKII) could be participants in mediating the contractile activity‐induced upregulation of PGC‐1*α* gene expression and activity.

### ROS are involved in contractile activity‐induced signaling, PGC‐1α expression, and PGC‐1α promoter activity

**Figure 3.**
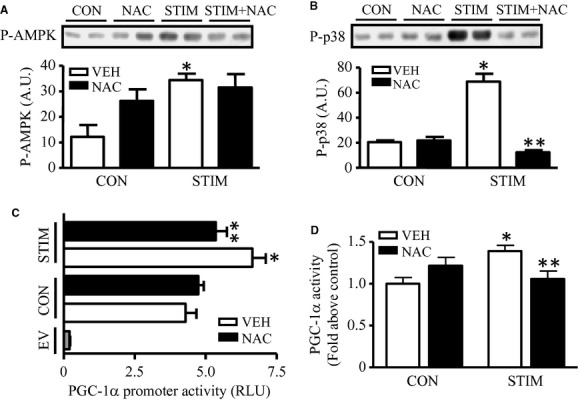
Effect of contractile activity‐evoked ROS signaling pathway on PGC‐1*α* gene expression. (A,B) Protein content of phosphorylated AMPK and p38 in control and stimulated cells with (*closed bar*) or without (*open bar*) the presence of 20 mmol/L N‐acetyl Cysteine (NAC). Representative Western blots of P‐AMPK and P‐p38 are shown (*top*), with the corresponding graphical quantification (*bottom*). Phosphorylated AMPK and p38 were corrected using total AMPK and p38 (not shown) as in Figure 2. A.U., arbitrary units, (*n* = 6). (C) Mouse PGC‐1*α* promoter was cloned into the pGL3 luciferase vector as previously described (Irrcher et al. 2009), cells were transfected with the indicated luciferase constructs and harvested for the measurement of luciferase activities. Transfection efficiency was normalized to renilla luciferase activity (pRL‐CMV; 5 ng/plate) (*n* = 6–8). (D) Cells were cotransfected with a chimeric gene encoding full‐length PGC‐1*α* fused to the DNA‐binding domain (DBD) of GAL4 of pBind vector, and a luciferase construct driven by five GAL4 DBD consensus sequences, referred to as pG5‐Luciferase vector. Graphical representation of PGC‐1*α* promoter activity (C) and PGC‐1*α* coactivation activity (D) expressed as luciferase activity from control and stimulated cells with or without NAC pretreatment (60 min) (*n* = 6–8). (*P < 0.05 vs. control vehicle cells; **P < 0.05 vs. stimulated vehicle cells). RLU, relative light units. CON, control; STIM, stimulation; VEH, vehicle; EV, empty vector).

To further determine the role of ROS on contractile activity‐induced kinase signaling and PGC‐1*α* gene expression, we preincubated myotubes with NAC (20 mmol/L), a powerful ROS scavenger, prior to contractile activity. As shown in Figure 3A and B, inhibition of ROS by NAC did not significantly affect the basal phosphorylation of AMPK or p38. This lack of effect of NAC on basal AMPK activity has been reported by us previously (Irrcher et al. 2009). NAC strongly inhibited the contractile activity‐induced increase in the phosphorylation of p38 (Fig. 3B), but this effect was not statistically significant for AMPK (*n* = 6 experiments; Fig. 3A). This suggests that the contraction‐induced increase in p38 activation is dependent on ROS production.

Contractile activity induced a 30–40% increase in PGC‐1*α* promoter activity above that found in quiescent cells (Fig. 3C). Although NAC had no effect on basal promoter activity in resting myotubes, it significantly blunted the increase in promoter activity due to contractile activity. Similarly, NAC also prevented the contractile activity‐induced increase in PGC‐1*α* activity. Thus, these data imply that ROS play an important role in mediating changes in PGC‐1*α* gene expression, and protein activity in the nucleus, during acute bouts of contractile activity.

### Ca^2+^ mediates basal and contraction‐induced CaMKII signaling and downstream effects on PGC‐1α promoter activation

**Figure 4.**
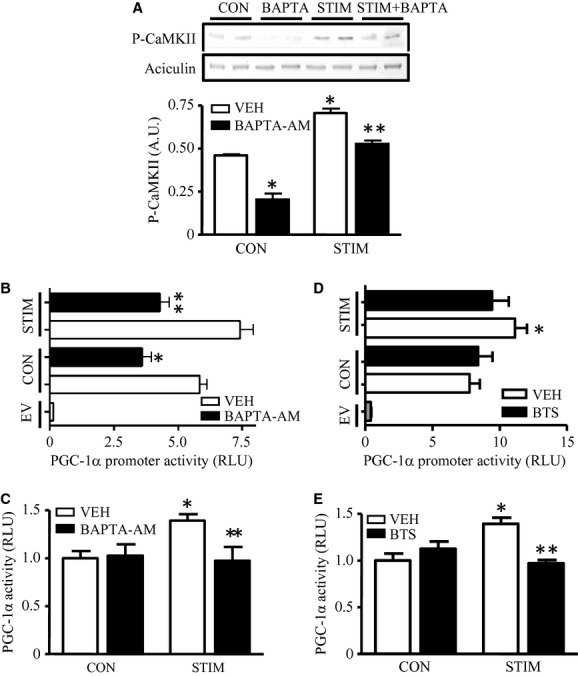
Effect of contractile activity on the Ca^2+^ signaling pathway and myosin ATPase activity. (A) Protein content of phosphorylated CaMKII in control and stimulated cells with (*closed bar*) or without (*open bar*) the presence of 100 *μ*mol/L BAPTA‐AM. Representative Western blots of P‐CaMKII are shown (*top*), with the corresponding graphical quantification (*bottom*). Phosphorylated CaMKII was corrected by its loading control. Graphical representation of PGC‐1*α* promoter activity (B) and PGC‐1*α* coactivation activity (C) expressed as luciferase activity from control and stimulated cells with or without BAPTA‐AM pretreatment (30 min). The effect on myosin ATPase inhibition on PGC‐1*α* gene expression was assessed through PGC‐1*α* promoter activity (D) and PGC‐1*α* coactivation activity (*E*) expressed as luciferase activity from control and stimulated cells with or without 150 *μ*mol/L N‐benzyl‐p‐toluene sulfonamide (BTS) pretreatment (30 min). (*P < 0.05 vs. control vehicle cells; **P < 0.05 vs. stimulated vehicle cells, *n* = 6–8). RLU, relative light units. CON, control; STIM, stimulation; VEH, vehicle; EV, empty vector).

We next investigated whether Ca^2+^ signaling could exert similar effects on PGC‐1*α* expression and activity during contractile activity. To do this, we preincubated myotubes with BAPTA‐AM (100 *μ*mol/L) prior to stimulation, a calcium chelator capable of preventing intracellular calcium increases (Connor et al. 2001). BAPTA‐AM also inhibits the contraction process and ATP turnover, but it does not interfere with muscle cell action potential propagation. BAPTA‐AM attenuated basal CaMKII phosphorylation, as well as PGC‐1*α* promoter activity in nonstimulated myotubes (Fig. 4A and B). As expected, increases in both PGC‐1*α* promoter activity (Fig. 4B), as well as PGC‐1*α* activity (Fig. 4C) brought about by contractile activity were abolished in the absence of increases in intracellular Ca^2+^. These data indicate that the inherently low intracellular Ca^2+^ levels found in quiescent muscle are important for signaling to PGC‐1*α* transcription at rest, and that the electrical activation and depolarization of the muscle are not sufficient signals to promote PGC‐1*α* expression and activity.

To evaluate whether contraction‐induced increases in intracellular Ca^2+^ were also important for signaling to PGC‐1*α*, we stimulated myotubes to induce contractile activity in the presence of BTS, an inhibitor of contraction at the level of myosin cross‐bridge formation (Shaw et al. 2003). Electrical stimulation induces Ca^2+^ release as normal, but contraction does not take place. Our results indicate that the normal contractile activity‐induced increase in PGC‐1*α* promoter activity, as well as transcriptional activity, was both attenuated in the presence of BTS, suggesting that Ca^2+^ release from sarcoplasmic reticulum plays a role in determining PGC‐1*α* expression during contractile activity (Fig. 4D and E).

### AMPK and p38 signaling kinase are important for acute contractile activity‐induced PGC‐1α gene expression

It has previously been shown that transcription of the PGC‐1*α* gene can be stimulated with AICAR treatment (Irrcher et al. 2008) or high levels of ROS (Irrcher et al. 2009). Other findings have also indicated that the activation of the p38 MAPK pathway by overexpression of MKK3E or MKK6E resulted in enhanced PGC‐1*α* promoter activity, which could be blocked by specific p38 inhibitors (Akimoto et al. 2005; Hong et al. 2011).

**Figure 5.**
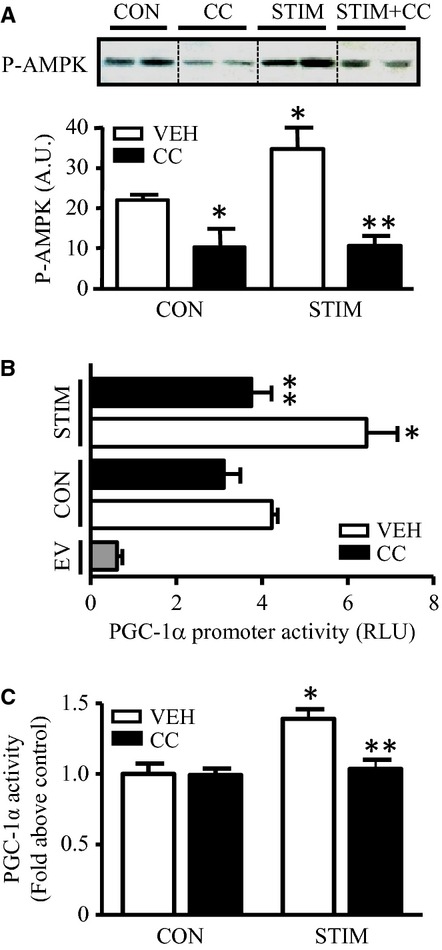
Contractile activity‐induced PGC‐1*α* gene expression is regulated by the phosphorylation of AMPK kinase pathway. (A) Protein content of phosphorylated AMPK in control and stimulated cells with (*closed bar*) or without (*open bar*) the presence of 40 *μ*mol/L Compound C (CC). Representative Western blots of P‐AMPK are shown (*top*), with graphical quantification (*bottom*). Phosphorylated AMPK was corrected by total AMPK as its loading control. The dashed lines between the bands indicate that the bands shown were derived from separate blots and spliced together to form a representative image of the data observed. (B, C) Graphical representation of PGC‐1*α* promoter activity (B) and PGC‐1*α* coactivation activity (C) expressed as luciferase activity from control and stimulated cells with or without AMPK pretreatment (30 min) (*n* = 6–8). (*P < 0.05 vs. control vehicle cells; **P < 0.05 vs. stimulated vehicle cells). RLU, relative light units. CON, control; STIM, stimulation; VEH, vehicle; EV, empty vector).

To gain further insight into the mechanisms of AMPK and p38‐mediated contraction‐induced PGC‐1*α* gene expression, C2C12 myotubes were pretreated with the AMPK inhibitor, Compound C (CC; 40 *μ*mol/L) or p38 inhibitor BIRB (10 *μ*mol/L), followed by contractile activity. CC treatment resulted in lower basal AMPK phosphorylation under quiescent conditions, and abolished the contractile activity‐induced increase in AMPK phosphorylation (Fig. 5A). The contractile activity‐induced increases in both PGC‐1*α* transcription and activity were also inhibited by CC (Fig. 5B and C). Notably, no significant changes were observed in PGC‐1*α* transcription or PGC‐1*α* activity under basal, resting conditions, suggesting that the activation of AMPK was not required for basal PGC‐1*α* transcription or its coactivation function.

**Figure 6.**
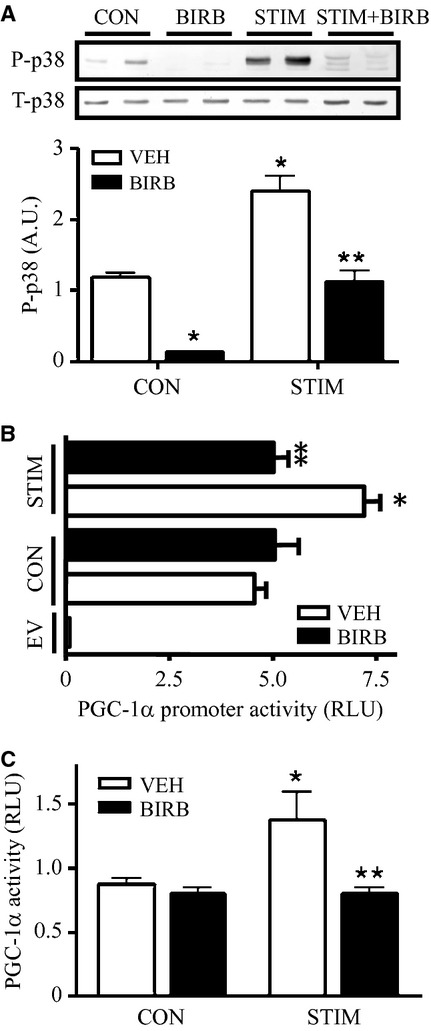
The involvement of activated p38 MAPK kinase in contractile activity‐induced PGC‐1*α* gene expression. (A) Protein content of phosphorylated p38 in control and stimulated cells with (*closed bar*) or without (*open bar*) the presence of 10 *μ*mol/L BIRB (BIRB796). Representative Western blots of P‐p38 are shown (*top*), with the corresponding graphical quantification (*bottom*). Phosphorylated p38 was corrected by total p38 as its loading control. *B and C:* Graphical representation of PGC‐1*α* promoter activity (B) and PGC‐1*α* coactivation activity (C) expressed as luciferase activity from control and stimulated cells with or without BIRB pretreatment (30 min) (*n* = 6–8). (*P < 0.05 vs. control vehicle cells; **P < 0.05 vs. stimulated vehicle cells). RLU, relative light units. CON, control; STIM, stimulation; VEH, vehicle; EV, empty vector).

Similar findings to those reported above were found with the inhibition of p38 with BIRB treatment. The phosphorylation of p38 was dramatically reduced under basal conditions, but was partially restored by contractile activity (Fig. 6A). The increases in PGC‐1*α* transcription and activity brought about by contractile activity were completely attenuated (Fig. 6B and C), but no effects of p38 inhibition on basal PGC‐1*α* transcription or promoter activity were noted.

## Discussion

It is well known that chronic exercise evokes mitochondrial alterations in skeletal muscle that have favorable physiological benefits with regard to metabolism and fatigue resistance during exercise (Hood 2001). An important contributor to this process is PGC‐1*α*, a transcriptional coactivator of multiple nuclear genes encoding mitochondrial proteins (NUGEMPs). PGC‐1*α* expression is known to be increased following acute (Baar et al. 2002; Pilegaard et al. 2003; Akimoto et al. 2005; Haase et al. 2011) and chronic (Adhihetty et al. 2003; Irrcher et al. 2003b) exercise. However, a complete understanding of the molecular mechanisms governing the expression and activity of PGC‐1*α* in response to individual bouts of exercise has not yet been fully elucidated. Therefore, the purpose of this study was to explore the role of acute contractile activity‐evoked signaling pathways involved in PGC‐1*α* expression and activity.

Using an established in vitro cell culture model of contractile activity (Connor et al. 2001; Irrcher et al. 2003a; Irrcher and Hood 2004; Joseph et al. 2004; Uguccioni and Hood 2011), we found that acute stimulation of myotubes resulted in a rapid but transient increase in PGC‐1*α* mRNA expression, suggesting a time‐dependent response to contractile activity. The underlying cause of PGC‐1*α* mRNA induction is likely linked to a transcriptional mechanism (Herzig et al. 2001; Czubryt et al. 2003; Daitoku et al. 2003; Akimoto et al. 2004, 2005), since direct measurements of PGC‐1*α* transcription indicated a coincident increase along with the mRNA. Since the increase in transcription was lower in magnitude than that of PGC‐1 mRNA, increases in mRNA stability may also be involved in the response to acute contractile activity. Accompanying these changes were increases in PGC‐1*α* protein translocation to the nucleus, and enhanced binding to DNA promoter regions (i.e., PGC‐1*α* protein activity), likely reflecting posttranslational modifications of the protein.

The enhanced PGC‐1*α* expression resulting from acute contractile activity was due to the activation of multiple signal transduction pathways. Within seconds of the onset of contractile activity, a number of rapid events occur that form part of the initial signaling process involved in PGC‐1*α* gene regulation, such as the change in membrane potential, elevations in cytoplasmic calcium (Ca^2+^), increases in ATP turnover, AMPK and p38 activation, and elevated ROS production (Cogswell et al. 1993; Irrcher et al. 2003a; Ojuka et al. 2003; Jorgensen et al. 2005). To identify the specific mechanisms involved in contractile activity‐mediated induction of PGC‐1*α* transcription in skeletal muscle cells, we transfected the mouse PGC‐1*α* promoter into myoblasts, allowed them to differentiate into mature skeletal muscle myotubes, and then subjected them to contractile activity in the presence or absence of inhibitors used to block specific signaling pathways. For example, to implicate p38 in this process, we employed BIRB796, which has been identified to inhibit all p38 isoforms in vitro and vivo (Kuma et al. 2005). Akimoto et al. (2005) have previously reported that contractile activity‐induced activation of the p38 pathway promotes PGC‐1*α* gene expression and skeletal muscle adaptations. In concert with these results, we observed a marked induction of p38 phosphorylation, as well as enhanced PGC‐1*α* transcription in response to 2 h of stimulation. Inhibition of p38 with BIRB significantly attenuated contractile activity‐induced PGC‐1*α* gene transcription, fortifying the importance of p38 MAPK kinase (Akimoto et al. 2005) in the regulation of PGC‐1*α* expression in response to contractile activity.

It has long been established that ROS play an important role in cellular functions because of their activation of signaling cascades, and ROS are increased in muscle by exercise or muscle contractions (Davies et al. 1982; Reid 2001; McArdle et al. 2005). Although the ROS signals that regulate PGC‐1*α* transcription are not yet fully understood, we have previously shown that ROS influence PGC‐1*α* mRNA expression via transcriptional activation of the PGC‐1*α* promoter. The mechanisms underlying this event can be accounted for, in part, by an overlapping GATA/Ebox sequence that binds USF‐1, an Ebox binding transcription factor (Irrcher et al. 2008, 2009). Our current results provide additional support for the importance of ROS produced during contractile activity. Two hours of contractile activity enhanced PGC‐1*α* promoter activity, an effect that was inhibited by pretreatment with NAC, a ROS scavenger. Interestingly, NAC completely abolished the contractile activity‐induced activation of p38, but not that of AMPK, suggesting that the contractile activity‐evoked PGC‐1*α* expression, as mediated by p38, is regulated by ROS production. Akimoto et al. (2005) have previously observed that MKK6E increases PGC‐1*α* promoter activity, as a direct result of activated p38 activity in myotubes.

In addition to the signaling role that ROS play, Ca^2+^ also contributes to contractile activity‐induced changes. Raising cytosolic Ca^2+^ has been shown to increase several markers of mitochondrial biogenesis, such as nuclear respiratory factor‐1 (NRF‐1) and 2 (NRF‐2) binding to DNA, as well as mitochondrial transcription factor A (Tfam) and PGC‐1*α* protein content (Freyssenet et al. 1999; Ojuka et al. 2003). We found that decreasing the concentration of Ca^2+^ by treating cells with BAPTA‐AM, a chelator of intracellular free Ca^2+^ (Shen et al. 2007), strongly inhibited the phosphorylation of CaMKII, the primary family of CaMK isoforms expressed in skeletal muscle. Thus, we suspect that the attenuated PGC‐1*α* gene expression in stimulated cells in the presence of BAPTA‐AM is linked to reduced CaMKII signaling. Alternatively, activation of cAMP‐responsive element‐binding protein (CREB) and activating transcription factor 2 (ATF2) can also occur through Ca^2+^ signaling. Activation of CREB stimulates its binding to cAMP response element (CRE) in the PGC‐1*α* promoter, thereby upregulating its expression (Akimoto et al. 2005; Yan et al. 2007).

Contractile activity is also a well‐known stimulus for AMPK activation, and this protein has been implicated in mediating PGC‐1*α* transcriptional activation (Akimoto et al. 2005; Irrcher et al. 2008). Previously, we have explored the use of AICAR, an AMPK activator, and found an increase in AMPK*α* phosphorylation at Thr^172^. This phosphorylation coincided with the associated changes in PGC‐1*α* gene transcription (Irrcher et al. 2008). In this study we used Compound C, a commonly used AMPK inhibitor, to further define the role of AMPK in PGC‐1*α* gene expression in stimulated cells. The inhibition of AMPK led to reductions in contractile activity‐induced PGC‐1*α* promoter activity. These data demonstrate that the inhibition of the AMPK kinase pathway is certainly also involved in contractile activity‐induced PGC‐1*α* gene expression. Thus, several redundant pathways exist which serve to activate PGC‐1*α* gene expression as a result of acute contractile activity.

**Figure 7.**
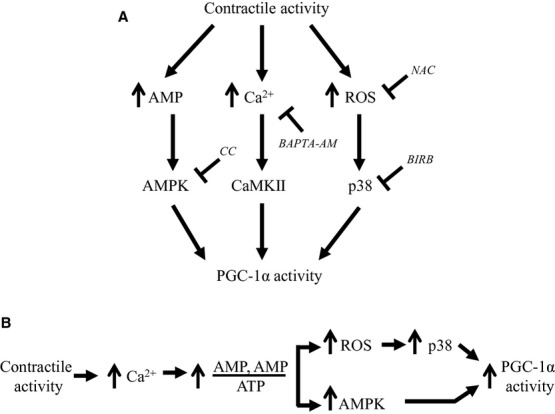
Hypothetical possibilities for the activation of PGC‐1*α* transcription and protein as a result of contractile activity. (A) Contractile activity activates parallel pathways which are all necessary for PGC‐1*α* transcription and/or activity. Inhibition or impairment of any of these pathways abolishes the exercise effect. (B) Contractile activity leads to a sequence of linear, ordered events which diverge as a consequence of increased mitochondrial respiration and ATP turnover. The subsequent increase in ROS and AMP kinase activity is both required for the full activation of PGC‐1*α* transcription and activity.

The regulation of PGC‐1*α* activity at the protein level during mitochondrial biogenesis can occur via changes in protein stability, subcellular localization or activation. PGC‐1*α* protein harbors an activation domain at the N‐terminus, as well as multiple LXXLL‐like motifs that allow for interactions with nuclear receptors (Lin et al. 2005). There are multiple sites within the primary sequence where posttranslational modifications can occur, allowing for the regulation of PGC‐1*α* activity. For example, the coactivator contains numerous p38 MAPK and AMPK phosphorylation sites (Puigserver et al. 2001; Jager et al. 2007), arginine methylation sites (Teyssier et al. 2005), sumoylation residues (Rytinki and Palvimo 2009), and several acetylation sites. We used a novel assay to determine PGC‐1*α* activity in skeletal muscle, involving the cotransfection of C2C12 cells with a plasmid encoding full‐length PGC‐1*α* fused to the DNA‐binding domain (DBD) of GAL4 of the pBIND vector and a luciferase construct driven by five GAL4 DBD consensus sequences. PGC‐1*α* has been shown to have intrinsic transcriptional activity when fused to the yeast GAL4‐DNA DBD (Puigserver et al. 1999; Knutti et al. 2000; Vega et al. 2000). Luciferase activity is a reflection of the ability of PGC‐1*α* to coactivate transcription factors, and thus has been defined as PGC‐1*α* protein activity. Here, we demonstrated that PGC‐1*α* protein activity was increased 35% by acute contractile activity. As with changes in PGC‐1*α* transcription, this increase in activity was abolished by inhibition of all of the contraction‐induced signaling pathways, including ROS, p38, AMPK, or CaMK. Thus, this indicates that multiple signal transduction pathways are required to activate PGC‐1*α* protein, and that nullification of any of these will reduce the contractile activity effect. An alternative possibility suggested by our data is the involvement of a linear sequence of events which occurs following contractile activity‐induced membrane depolarization, involving an increase in cytosolic Ca^2+^, cross‐bridge cycling, and ADP‐induced respiration. Subsequently, the rise in ROS formation due to respiration could activate p38, thereby triggering PGC‐1*α* protein activity. In parallel, the increase in free ADP also leads to a rise in AMP concentration, which activates AMPK, and subsequent PGC‐1*α* protein activity. These two possibilities are illustrated in Figure 7.

In resting muscle, our data suggest that p38, AMPK, or ROS do not contribute to basal levels of PGC‐1*α* promoter activity. In contrast, reductions of intracellular Ca^2+^ levels in resting muscle significantly attenuated the activity of the promoter. These results indicate that basal levels of PGC‐1*α* transcription are more sensitive to resting Ca^2+^ levels, compared to ROS, p38 or AMPK signaling. However, PGC‐1*α* activity was not influenced by the low activity of these selected signaling pathways in resting muscle, suggesting that alternative events (i.e., de‐acetylation) may be more important for maintaining PGC‐1*α* protein activity in resting muscle.

Taken together, it appears that activation of the signaling molecules AMPK, ROS, and Ca^2+^ is necessary for the regulation of contractile activity‐induced PGC‐1*α* gene expression, governed partly through the p38 MAPK and CaMKII pathways. The influence of these pathways, with the exception of Ca^2+^, was not apparent in resting muscle, but became evident under contractile activity‐induced conditions, revealing their involvement in the regulation of PGC‐1*α* transcription and DNA‐binding activity during “exercise”. Whether these signaling pathways are arranged as a linear sequence of events or as largely independent pathways (Fig. 7) during contractile activity is still under investigation.

## Acknowledgments

Y. Zhang was a recipient of a China Scholarship Council award. D.A. Hood is the holder of a Canada Research Chair in Cell Physiology.
